# A density functional theory study of tyrosine‐proton mediated transport in Ag‐filamentary nanodevices

**DOI:** 10.1002/smo2.70019

**Published:** 2025-09-03

**Authors:** Dan Berco

**Affiliations:** ^1^ School of Electrical Engineering & Computer Science Washington State University Pullman Washington USA

**Keywords:** Ag‐filamentary memristors, amino‐acid devices, Hydrogen sensors, neuromorphic computing, proton‐mediated charge transport, tyrosine‐based nanostructures

## Abstract

The development of electronic circuits designed to emulate the functionality of biological neural networks has increased significantly in recent years. Specifically, memristor‐based neuromorphic operation has been demonstrated using various material combinations. One class of devices replicates the ion‐concentration‐gradient buildup that precedes neurotransmitter release in biological synapses. Some of these devices incorporate amino‐acid‐rich solutions as an active layer. This work presents a density functional theory study of such a device. The interaction between an Ag‐filamentary memristor and different Hydrogen concentrations in a tyrosine‐rich environment was evaluated. Two mutually exclusive structures were studied, and the resulting source‐to‐drain currents were compared with experimental observations. One structure was based on Tyrosine‐H blocks linked to Ag atoms as a charge conduction path, while the other placed these blocks in parallel with Ag partial filaments between the source and drain. The results indicate that the second aligns with experiments and supports the hypothesis that tyrosine can act as an enabler for proton‐mediated charge transport. Furthermore, the insights into the electronic transport properties of specific molecules can provide a theoretical background for designing advanced Hydrogen sensors and amino acid detectors.

## Graphical abstract

A density functional theory study of tyrosine and proton‐assisted transport in an Ag‐based nanodevice is presented. The study confirms the hypothesis that tyrosine molecules enable proton‐mediated charge transport in Ag conductive filaments. Both relative tyrosine–Ag atom placement and proton concentrations were investigated. The simulated current results align with the experimental observations.

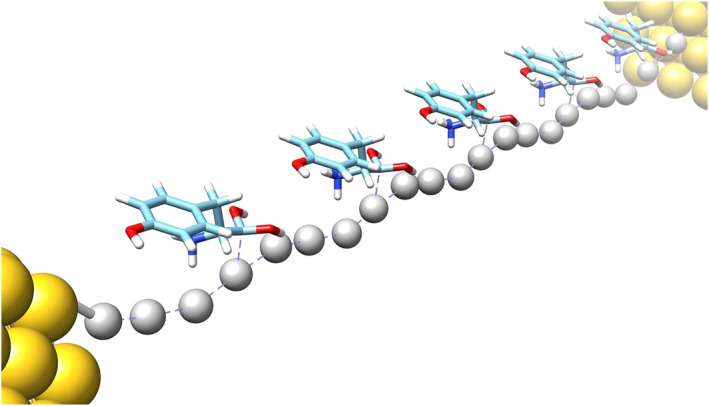

## 1 INTRODUCTION

In the early 1990s, Shibata and Tadahiro^[1]^ fabricated a transistor with gate‐level weighted sum using metal‐oxide‐semiconductor (MOS) technology. Lee et al.^[2]^ demonstrated an analog floating‐gate synapse for general‐purpose neural computation. At a retrospective level, these groundbreaking devices preceded the artificial intelligence revolution by decades. Plenty of solid‐state devices mimicking biological neurons have been demonstrated since then. Additionally, the exploration of memristors[^3^, ^4^] as artificial synapses gained significant traction due to the reversible resistive switching effect.^[5]^ Memristors can exhibit analog, non‐volatile properties, and even alternating current rectification,^6^, ^7^ and may be used to implement hardware‐based artificial neural networks (HNN). They offer a notable advantage over software‐based implementations, especially in terms of processing speed. The most common method for implementing them is based on perceptron networks.[^8^, ^9^]

Artificial synaptic devices aim to imitate the physiology of biological synapses. These devices form the foundation for numerous HNN implementations, including those based on field‐programmable gate arrays (FPGAs),[^10^, ^11^, ^12^, ^13^, ^14^] application‐specific integrated circuits (ASICs),[^15^, ^16^] and bio‐inspired robotic vision.[^17^, ^18^, ^19^, ^20^, ^21^] However, the operation of biological synapses is complex and involves chemical interactions and pH changes, in addition to ionic charge transfer.^[22]^ A key process is proton concentration buildup before neurotransmitter signaling. Protons effectively turn on the synapse prior to neurotransmitter release. This process, known as proton‐mediated signaling, involves the activation of ionic channels. Proton‐coupled electron transfer, where protons serve as charge transfer mediators,[^23^, ^24^] plays a crucial role in signal activation.^[25]^

More recently, Kwon et al. demonstrated different devices using peptide materials.^[26]^ They fabricated tyrosine‐rich YYACAYY (Y7C) structures. These peptides can form a helix dimer and spontaneously assemble into a 2‐D film.^[27]^ The group concluded that tyrosine could serve as a catalyst for metal ion redox reactions even in thick films. Such peptide‐based devices may be used in various applications, including bimodal memory,^[26]^ degradable memristors,^[27]^ humidity sensors,^[28]^ synaptic transistors,^[29]^ and analog resistive switches.[^30^, ^31^] One common feature they share is that their tyrosine‐rich peptide medium allows for proton‐driven activation and mediation of charge transport. This results in both resistive switching, for a synapse‐like behavior, and humidity sensing capability‐effectively replicating proton‐mediated signaling at biological synapses within a proton‐modulated active layer.^[32]^

This work features a density functional theory[^33^, ^34^] (DFT) study of a two‐terminal nanodevice that models an Ag‐filamentary memristor with tyrosine molecules. Charge transport was simulated under different Hydrogen (H) environmental concentrations, and source‐to‐drain bias (*V*_DS_) was simulated. The research used two mutually exclusive structures, and the resulting currents were compared to experimental data. One structure featured tyrosine‐H blocks linked to Ag atoms forming a charge conduction pathway, while the other placed those blocks in parallel with Ag partial filaments between the source and drain. The findings indicate that the source‐to‐drain current (*I*_DS_) in the second structure aligns with the experimental results. The manuscript first introduces the architectural concept and structural relaxation, then presents current calculations, followed by quantum transport spectral components for each structure.

## 2 RESULTS AND DISCUSSION

### 2.1 Architectural concept

Hydrogen plays an important role in chemical and biological processes through mechanisms involving proton‐coupled electron transfer (PCET). In the form of protons (H^+^), Hydrogen can modulate the reactivity and pathways of reactions by altering Molecular orbitals (MO). Protonation can change the electron density of molecules, assisting or impeding electron transfer. A PCET reaction involves the transfer of a proton and an electron. Tyrosine radicals in enzymes often undergo PCET, and Hydrogen bonds act as proton relays in PCET. Localized electric fields affect PCET rates, and Hydrogen can thus tune chemical processes to be energetically favorable through proton availability.

**FIGURE 1:**
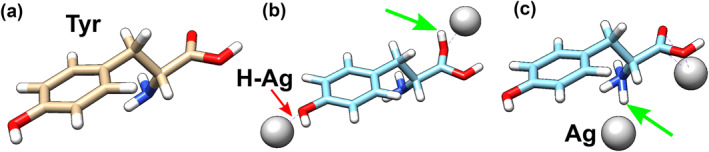
Stick diagram representations of building blocks used to construct both structures under simulation. (a) Tyrosine molecule. (b) A single tyrosine molecule and a single H (green arrow) as building blocks for the **first** structure. Two Ag atoms (silver spheres) are positioned at either end of the charge transport path (*z*‐axis). (c) A single Tyrosine molecule and a single H (green arrow) as a building block for the **second** structure. Two Ag atoms (silver spheres) are placed in parallel. The Ag atoms represent part of a filamentary segment for charge transport along the *z*‐axis.

Transport calculations in this work were performed on two different structures. Each structure formed a nanodevice with two conductive terminals, one acting as a source and the other as a drain. A charge transport central region was placed between these terminals along the *z*‐axis. In each case, the central region incorporated Ag atoms along with Tyrosine‐based building blocks. These elementary building blocks are shown in Figure 1. A detailed overview of the structures is provided in the next section. All the building blocks contained a single tyrosine molecule and an H atom. Ag atoms were incorporated into the central region in various placements relative to the building blocks.

In the first structure, an H atom bonded to a tyrosine molecule interacts with Ag atoms to form a charge transport path. In the second structure, Ag‐based partial filaments create the main conductive path, while Tyrosine‐H blocks are positioned adjacent to them. This setup was designed to evaluate proton‐assisted transport properties. Regarding charge transport from source to drain, the structures are said to be orthogonal to each other because the first transport path involves tyrosine molecules and the second does not. A stick diagram of a standalone Tyrosine is shown in Figure 1a. Figure 1b displays a tyrosine‐H building block in series (relative to charge conduction along the *z*‐axis) with two Ag atoms. Figure 1c illustrates a Tyrosine‐H building block in parallel (relative to charge conduction) with two Ag atoms.

The two structures were designed to be mutually exclusive concerning charge transport along the main lateral symmetry line from source to drain (*z*‐direction). Their main difference is concerning quantum charge transport, which is most likely to occur in this direction. In the first structure, five tyrosine molecules form an integral part of this pathway. The molecules were arranged to ensure that any current flows through conduction channels induced by their structure. Their spatial configuration was chosen so that their principal axis aligned with this pathway. Conversely, conduction in the second structure relies on partial Ag filaments. Physically placing tyrosine molecules near such filaments was intended to assess their supportive role to the overall current.

Hydrogen placement was based on similar principles. In the first case, single H atoms were positioned between tyrosine molecules, and in the second, as an intermediary agent between a single molecule and a silver filament. In both scenarios, their locations were chosen to maximize wave function overlap with an adjacent tyrosine molecule. Both structures were allowed to relax and reach equilibrium. After relaxation, conduction channels were evaluated, and the total current was estimated as a function of drain‐to‐source bias and H concentration. Finally, this current‐voltage dependency was compared with experimental data.

### 2.2 Structural relaxation

**FIGURE 2:**
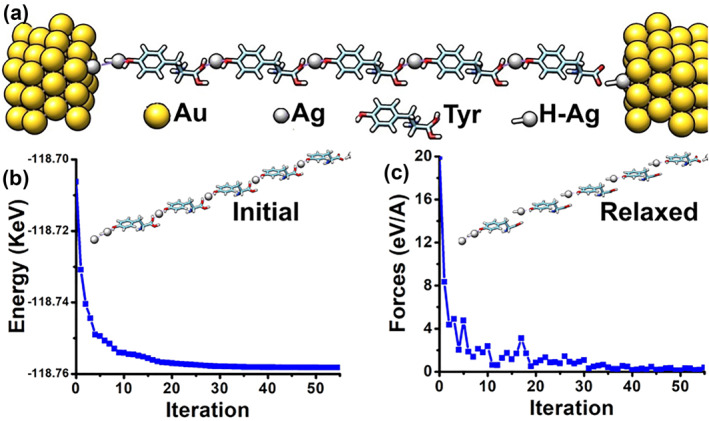
Relaxation of the **first** structure. (a) Schematic illustration of the first structure: two Au clusters serve as the source and drain electrodes on the left and right sides; five serial Tyrosine‐H blocks constitute the charge transport layer with Ag placed between them. (b) Overall energy as a function of iteration steps. (c) Total forces as a function of iteration steps. *Insets*: the positions of Ag atoms and Tyrosine‐H molecules in the initial and relaxed configurations.

**FIGURE 3:**
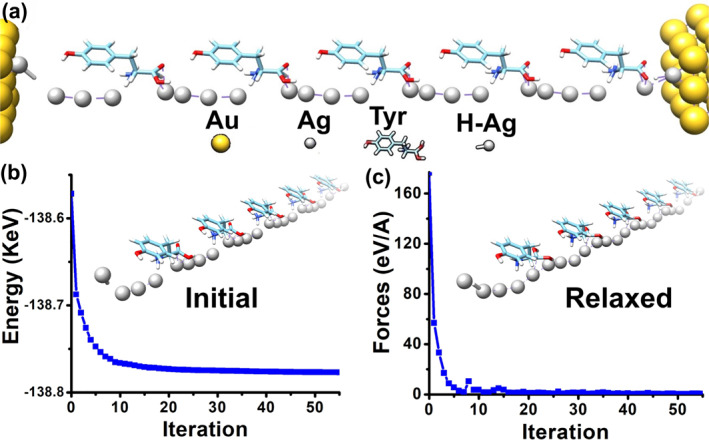
Relaxation of the **second** structure. (a) Schematic illustration of the second structure: two Au clusters serve as the source and drain electrodes on the right and left sides; five Ag partial filaments alongside Tyrosine‐H molecules form the charge transport layer. (b) Overall energy as a function of iteration steps. (c) Total forces as a function of iteration steps. *Insets*: the positions of Ag atoms and Tyrosine‐H molecules in the initial and relaxed configurations.

This section covers device construction and structural relaxation. Each nanodevice had Au‐based source and drain regions along with a central charge transport region. Both structures shared the same physical dimensions. The source and drain were 8.65 Å wide (*x*‐axis), 6.7 Å thick (*y*‐axis), and 7.1 Å long (*z*‐axis). The central region had the same width and thickness, with a length of about 81.1 Å along the *z*‐axis. The total device length was approximately 95.3 Å. The central region was built using five sequential building blocks in each case. In the first case, the blocks were arranged in series with Ag atoms along the charge transport path (Figure 2a). In the second structure, tyrosine‐H blocks were placed adjacent to Ag filaments, as shown in Figure 3a.

Once an initial placement was made, the devices underwent a structural relaxation process. Such relaxations rely on time–domain simulation that executes molecular dynamics algorithms. This relaxation ends at an equilibrium point of the microcanonical ensemble where both energy and forces in the system are minimized. The classical equation of motion for a system with *N* particles is (indexed as *i* = 1, 2 … *N*):

(1) $$m_{i}\frac{d^{2}{\vec{r}}_{i}}{dt^{2}}={\vec{f}}_{i}$$

with *m* the mass, *f* the force, and *r* the location vector. If the particles are interacting through a potential *v*, the Hamiltonian can be written with the momentum *p* as follows:

(2) $${\dot{\vec{p}}}_{i}=-\nabla_{{\vec{r}}_{i}}v={\vec{f}}_{i}$$

Conservation laws determine the time evolution of the system. With the Hamiltonian *H*, we can write:

(3) $$\frac{dH}{dt}=\frac{\partial H}{\partial t}+\frac{\partial H}{\partial \vec{p}}\frac{\partial \vec{p}}{\partial t}+\frac{\partial H}{\partial \vec{q}}\frac{\partial \vec{q}}{\partial t}=0$$

Calculating the time‐dependent trajectories involves either solving a set of 3N second‐order differential equations or a set of 6N first‐order equations. In either case, the system may be considered relaxed once the total energy is minimized and the sum of all forces falls below a predetermined threshold. The final configuration is the optimized device geometry used in subsequent stages. In this work, the maximum force limit was set to 0.05 eV/Å. The maximum step length for relaxation was set to 0.2 Å.

Figure 2 illustrates the relaxation process of the first structure. Figure 2a details the shape and elements used to construct the device. Two Au electrodes are shown on the right and left sides, serving as source and drain terminals. The central region between them consists of five building blocks (as shown in Figure 1b) placed consecutively. The simulation reaches a steady state after about 55 steps when minimizing the total energy, as shown in Figure 2b. Figure 2c shows that the total forces drop below the set maximum value.

Figure 3 illustrates the relaxation process of the second structure. In this case, the device shown in Figure 3a was constructed using five building blocks, as seen in Figure 1c. These blocks were placed next to five Ag partial filaments. As before, the structure reached an optimized state after approximately 55 simulation steps. The total energy is shown in Figure 3b, and the total forces are displayed in Figure 3c.

### 2.3 Proton concentration dependent current

The source‐to‐drain current *I*_DS_ as a function of H concentration for both structures is analyzed in this section. H atoms at various locations were removed, and the device's current was simulated at each step. Since there are a total of five Tyrosine blocks in the central region, the removal of a single H can be considered as a 20% reduction in ambient concentration. All calculations were based on the built‐in linear combination of atomic orbitals[^35^, ^36^] (LCAO) calculator with an electrode temperature of 300 K. In molecular orbital theory, this concept describes the way atomic orbitals combine to form MO. These orbitals are detailed by a basis set that is an integral part of the DFT software.^[37]^ MO are formed by taking linear combinations as weighted sums of these atomic orbitals to form the overall function *Ψ* according to the following equation:

(4) $$\Psi_{MO}=\sum_{i=1}^{N}c_{i}\varphi_{i}$$

with *i* = 1.*.N* elements, *c*_*i*_ the corresponding weight, and *φ*_*i*_ the individual orbital. In this work, both single‐zeta and double‐zeta polarized basis sets were used. Zeta (*ζ*) refers to the number of functions used to describe each atomic orbital. *I*_DS_ as a function of *V*_DS_ is calculated using the Landauer–Büttiker formula:

(5) $$I_{DS}=\frac{2e}{h}\int_{-\infty }^{\infty }T_{\left(E,V_{DS}\right)}\left|f_{S\left(E\right)}-f_{D\left(E\right)}\right|dE$$

where $T_{\left(E,V_{DS}\right)}$ is the transmission probability of an electron at energy *E* and bias *V*_DS_, and *f*_*S*_ and *f*_*D*_ are the Fermi‐Dirac distributions for source and drain, respectively.

**FIGURE 4:**
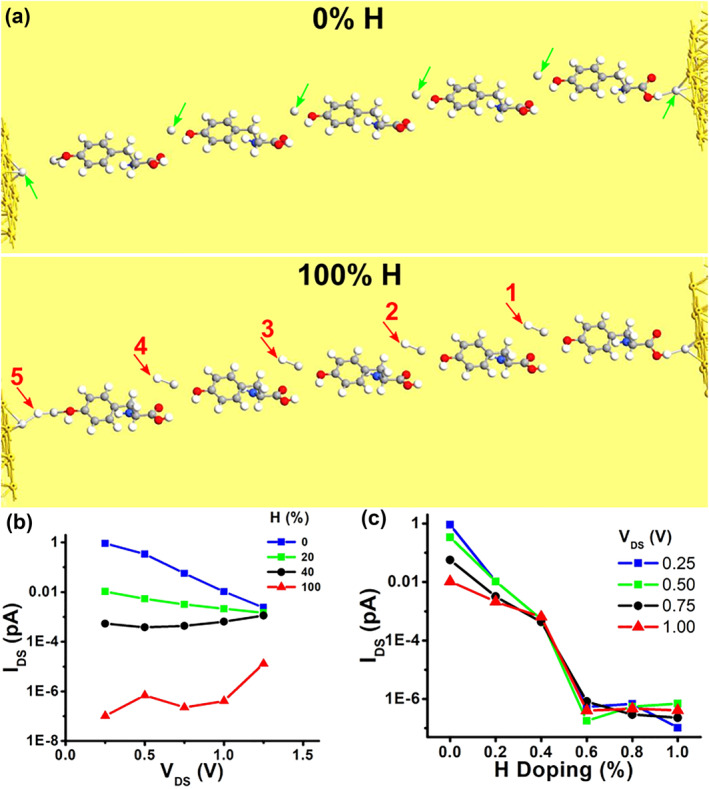
Device source‐to‐drain current as a function of ambient H concentration for the **first** structure. (a) Schematic diagram of the first structure at 0% and 100% H concentrations; the green arrows indicate Ag atoms, and the red arrows indicate H with adjacent Ag. (b) Source‐to‐drain current (*I*_DS_) as a function of source‐to‐drain voltage (*V*_DS_) at different H concentrations. (c) Source‐to‐drain current (*I*_DS_) as a function of H concentration at various *V*_DS_ levels.

Figure 4a shows a schematic of the central region of the first structure with 0% and 100% concentrations. The green arrows point to Ag atoms, whereas the red arrows indicate H atoms. For a 0% concentration, no H atoms are present near the Tyrosine molecules. Figure 4b illustrates *I*_DS_ as a function of the drain‐to‐source bias *V*_DS_ for different concentrations. The current *I*_DS_ decreases as the concentration increases. The maximum calculated current for this structure is around 1 pico‐ampere. At a concentration of 100%, *I*_DS_ drops to a background leakage level that is six orders of magnitude lower. This level remains relatively stable regardless of the bias level. A possible reason is that the passivation of Ag atoms eliminates transmission states through the structure. This is supported by the results in Figure 4c, which show *I*_DS_ as a function of H concentration for different *V*_DS_. *I*_DS_ quickly drops to this background leakage level once the concentration exceeds 50%.

**FIGURE 5:**
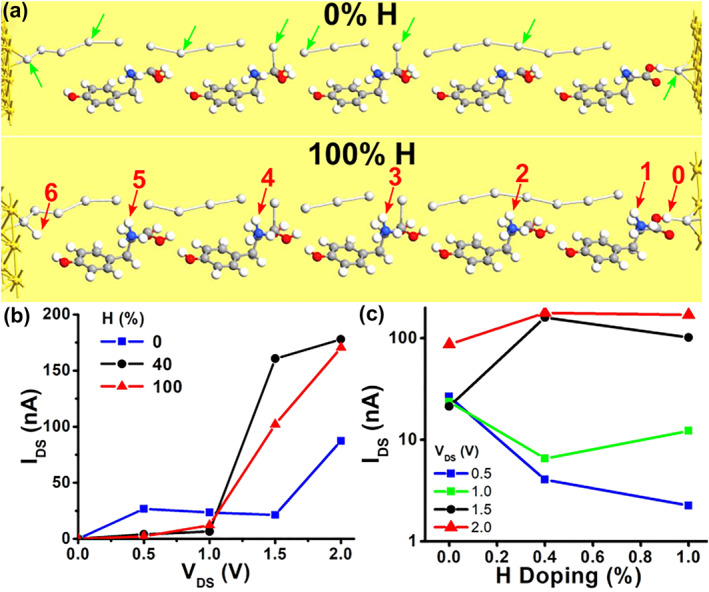
Device source‐to‐drain current as a function of ambient H concentration for the **second** structure. (a) Schematic diagram of the second structure at 0% and 100% H concentrations; the green arrows indicate Ag partial filaments, and the red arrows indicate H bonded to tyrosine molecules. (b) Source‐to‐drain current (*I*_DS_) as a function of source‐to‐drain voltage (*V*_DS_) at different H concentrations. (c) Source‐to‐drain current (*I*_DS_) as a function of H concentration for different *V*_DS_.

The simulation results for the second structure are shown in Figure 5. As mentioned earlier, tyrosine molecules with bonded H are placed adjacent to Ag partial filaments (Figure 5a). Compared to the previous results, the main difference is the maximum current. In this case, *I*_DS_ is in the order of hundreds of nano‐Amperes, which is roughly five orders of magnitude larger and conforms to experimental findings. Furthermore, *I*_DS_ as a function of the source‐to‐drain bias *V*_DS_ for different concentrations (Figure 5b, the 20% plot is omitted due to convergence issues) displays a resistive conduction as expected from experimental observations of filamentary memristors. Furthermore, Figure 5b,c show current increase with both *V*_DS_ and H concentration. *I*_DS_ increased by up to two orders of magnitude when *V*_DS_ increased from 1.0 to 2.0 V (Figure 5b). In addition, *I*_DS_ increased by the same ratio as the concentration increased from 0% to 100% in Figure 5c. The results show a current increase with H concentration for any given bias. The higher current and transmission channels can be attributed to the partial silver filaments that are conductive. These results are consistent with the proton recharge behavior observed by Kwon et al.^[32]^ and support the hypothesis that charge transport in the device is indeed proton‐mediated.

Specifically, they demonstrated that the source‐to‐drain current is roughly within the same order of magnitude for the Au‐electrode Y7C peptide device, considering humidity. Figure 4c shows that the current drops by about 6 orders of magnitude as H concentration increases. This indicates that the first structure is inconsistent with these observations. Figure 5c shows that the current varies by less than one order of magnitude. For a larger *V*_DS_, current changes are even smaller, as observed by Kwon et al.^[32]^ Moreover, the current drop associated with increased humidity (Figure 5c, *V*_DS_ ≥ 1.5 V) is consistent with their observations for vacuum and humid air ambience. The second structure is therefore in better agreement with the experimental observations.

Protons located near silver filaments associate with Ag atoms by inducing a local electric field and thereby affecting the overall conductance. Moreover, they can facilitate hopping sites for electron conduction. Hydrogen can also adsorb on such atomic‐scale filaments, thus changing their electronic structure. Shifting the Fermi level and electrostatic potential will affect transport properties and can account for the current behavior in the second structure as concentrations change.

### 2.4 Projected device density of states

Density functional theory, together with non‐equilibrium Green's function formalism,^[38]^ can be used to evaluate the projected device density of states (PDDOS) as the energy‐resolved spectral density of the central scattering region under bias. It identifies which atoms and associated orbitals add conduction channels and bias‐induced changes in orbital energies. By adding the contributions from the source and drain electrodes, we can obtain the spectral density matrix *ρ*:

(6) $$\rho_{\left(E\right)}=\rho_{S\left(E\right)}+\rho_{D\left(E\right)}$$

with *S*_*ij*_ as the orbital overlaps, the total PDDOS *D*_*(E)*_ is written as follows:

(7) $$D_{\left(E\right)}=\sum_{ij}\rho_{ij\left(E\right)}S_{ij}$$

**FIGURE 6:**
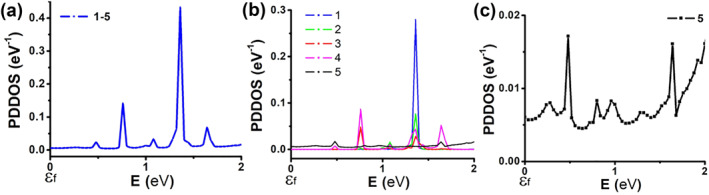
Projected device density of states for H atoms (according to their locations as specified in Figure 4) in the charge transport region of the **first** structure (Fermi level marked as *Ɛ*_*f*_). (a) Summation over all H states in the central region. (b) Contribution of individual H_1–5_. (c) The contribution of H_5_ resulting in a very low background current.

The PDDOS calculations for the first structure are shown in Figure 6. The figure presents the added contribution of individual H atoms associated with specific building blocks, numbered 1–5, as shown in Figure 4a. It helps identify specific conduction channels and thus the contribution of each H to the total current. The total PDDOS of all H atoms is shown in Figure 6a, with individual contributions in Figure 6b. Figure 6c displays the contribution of H_5_ alone. The figure indicates that the main contribution at approximately 1.4 eV comes from H_1_, while the H_3_ and H_4_ add states are at around 0.75 eV. H_5_ contributes a few states across all energy ranges. This contribution may be associated with the background leakage discussed earlier.

**FIGURE 7:**
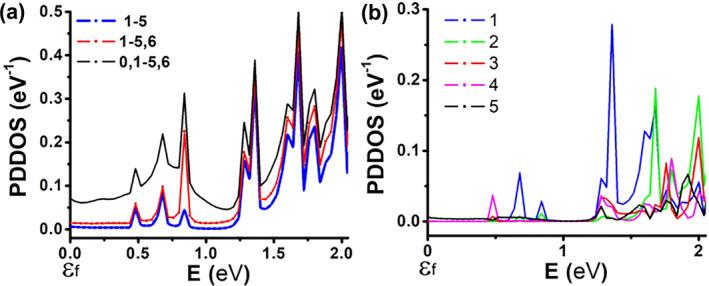
Projected device density of states for H atoms (according to their locations as specified in Figure 5) in the charge transport region of the **second** structure (Fermi level marked as *Ɛ*_*f*_). (a) Summation over all H states for the central region; the total contribution is separated into three plots showing the added effects of atoms H_0_ and H_6_ to the summation over H_1–5_. (b) Individual contribution of atoms H_1–5_.

Figure 7 shows the overall PDDOS of the second structure. The figure displays the combined contribution of individual H atoms associated with specific building blocks, numbered 0–6, as shown in Figure 5a. Figure 7a illustrates the total contribution of H_1–5_, along with the additional contribution of H_0_ and H_6_. In this case, H_0_ influences PDDOS at all levels. Referring to Figures 4 and 5a, in both cases, an H atom is connected between one terminal and the central region, and forms a stepping stone for charge transport. Therefore, observing a background current in similar structures can indicate the existence of such intermediate H.

Figure 7b details the individual PDDOS contributions of H_1–5_. As before, H_1_ contributes mostly at an energy of ∼1.3 eV. However, H_3_ and H_4_ are not as dominant around 0.75 eV in this case. A key difference here is the existence of significant and continuous states above 1.3 eV. This can explain the spike in current and resistive behavior seen in Figure 5b. Since H_1_ contributes mostly to this current step, calculating the derivative of *I*_DS_ in actual devices may imply the existence of H near the source terminal. Background leakage can indicate the existence of H along the charge transport path (H_0_).

### 2.5 Transmission spectrum

**FIGURE 8:**
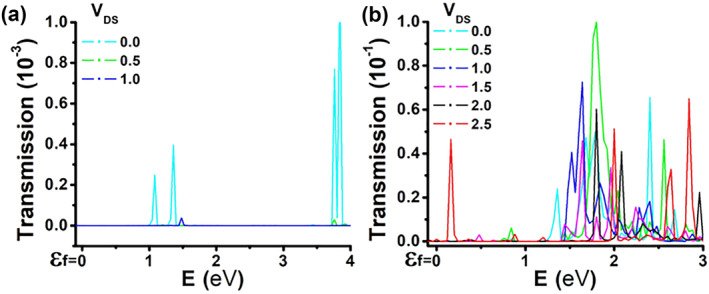
Summation over the transmission eigenvalues from source‐to‐drain as a function of energy for different bias levels (Fermi level marked as *Ɛ*_*f*_). (a) **First** structure. (b) **Second** structure.

The complete transmission spectrum (unitless value indicating probability) as a function of energy and bias is shown in Figure 8. In each case, the transmission is calculated for a 100% concentration and includes all H contributions. Both figures are plotted over similar axis ranges for easier comparison. The spectrum plots provide a better understanding of the energy levels that contribute to the overall current at different bias voltages. Figure 8a shows the spectrum of the first structure, and Figure 8b shows the second one. A key difference between the two is the abundant transmission channels in the second structure at energies above 1.5 eV. Additionally, the overall transmission in the second case is two orders of magnitude higher and explains the significantly larger *I*_DS_ observed in Figure 5b. Furthermore, the relatively stable *I*_DS_ for *V*_DS_ = 2.5 V seen in Figure 5c, regardless of doping level, can be explained by the peak (red) next to the Fermi level in Figure 8b. This suggests that some transport can occur at room temperature.

### 2.6 Positional transmission amplitude

**FIGURE 9:**
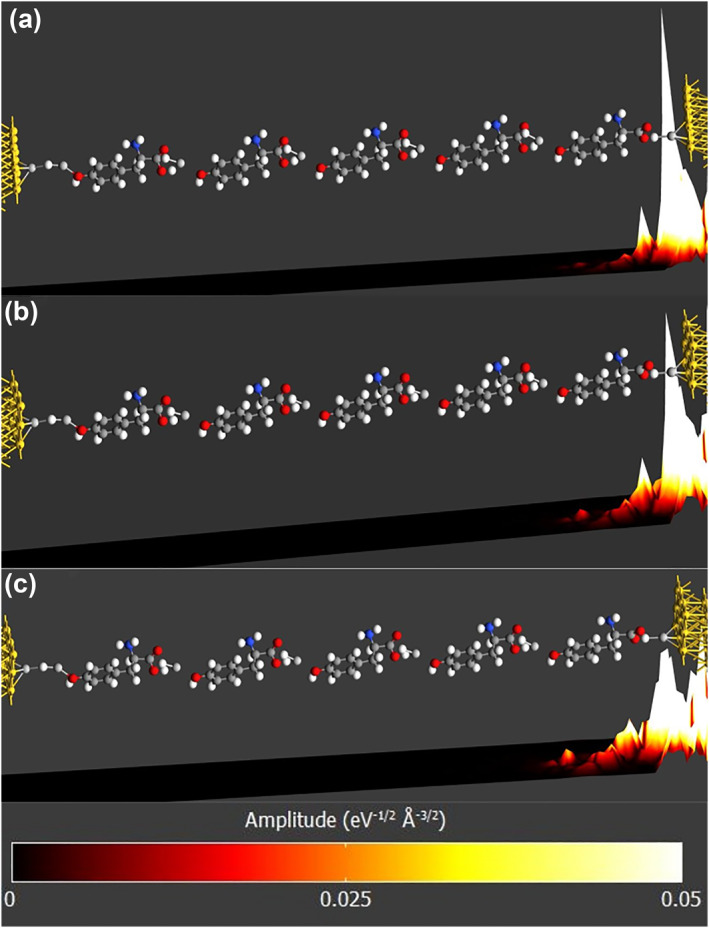
Projected amplitude of the dominant transmission mode along the charge transport *z*‐axis of the **first** structure. (a) *V*_DS_ = 0 V. (b) *V*_DS_ = 1.0 V. (c) *V*_DS_ = 2.0 V.

This section demonstrates the amplitude of the dominant transmission eigenstate along the central region for each structure. These visualizations help to show the contribution and impact of individual building blocks along the charge transport path. Figure 9 displays the 3D amplitude plot from source to drain at different *V*_DS_ levels for the first structure. Figure 9a shows the base amplitude at zero bias. The primary contribution comes from Ag and H atoms located on the source side. This is expected because they act as a bottleneck for charge transport. As *V*_DS_ increases, the amplitude of Tyrosine‐H blocks closer to the source becomes more noticeable. However, the overall spectrum remains small, so the amplitude is still relatively low. The increased contribution to the amplitude, with higher bias levels, is associated with the emerging peaks in Figure 9b,c.

**FIGURE 10:**
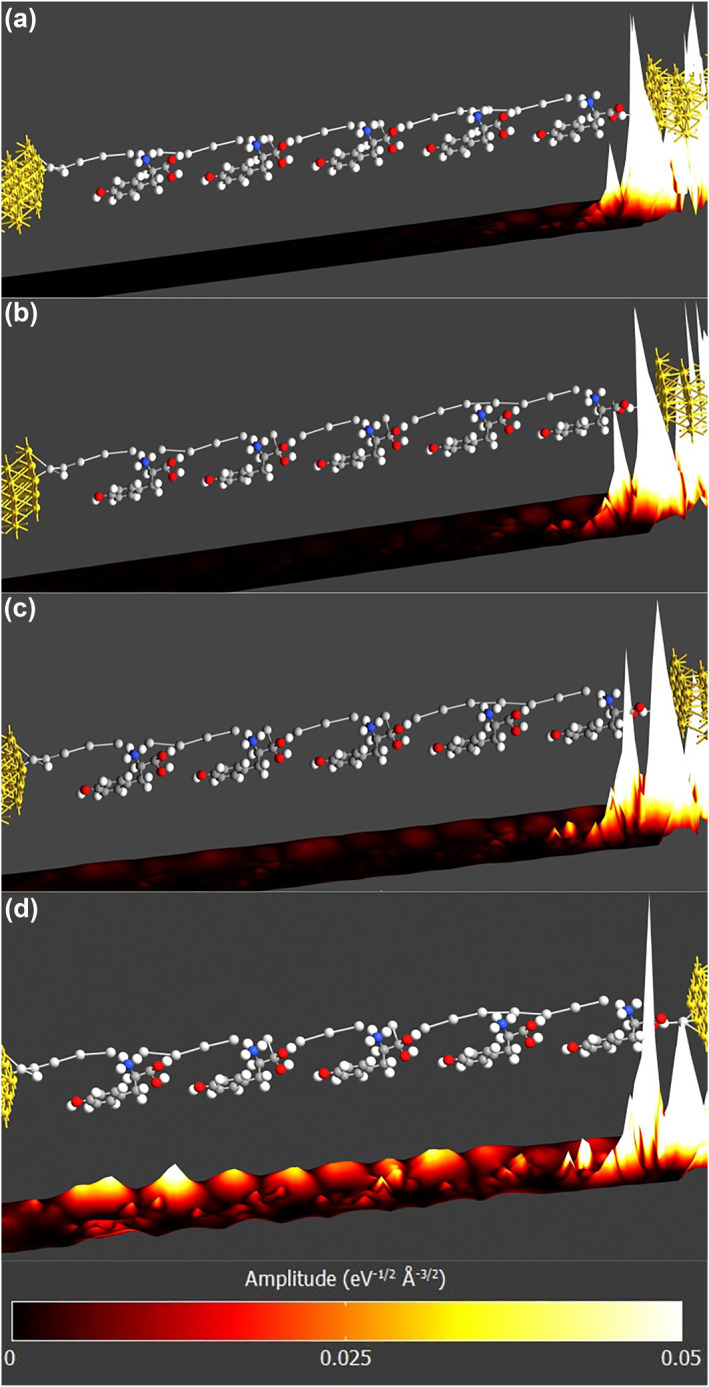
Projected amplitude of the dominant transmission mode along the charge transport *z*‐axis of the **second** structure. (a) *V*_DS_ = 0 V. (b) *V*_DS_ = 1.0 V. (c) *V*_DS_ = 2.0 V. (d) *V*_DS_ = 2.5 V.

Figure 10 displays the 3D amplitude plot from source to drain at different *V*_DS_ levels, along the second structure. Figure 10a gives the base amplitude with zero biasing. As before, the main contribution comes from Ag and H located directly at the source. However, in this structure, the contribution of blocks further from the source to the transmission becomes more dominant as the bias is increased, as shown in Figure 10b–d. For *V*_DS_ > 2.0 V, transmission occurs throughout the entire structure. This aligns with the spectrum seen in Figure 8b and indicates that transport can occur in these levels via abundant channels that become available.

## 3 CONCLUSIONS

In summary, this work presented a density functional theory study of the interaction between an Ag‐filamentary memristor and varying proton concentrations in a tyrosine‐rich environment. Two mutually exclusive structures were simulated, and the resulting currents were compared to experimental data. The first structure featured Ag and Tyrosine‐H blocks as a charge conduction path, while the second had them arranged in parallel with Ag partial filaments between the source and drain. The results suggest that the second configuration aligns with experimental observations and supports the hypothesis that Tyrosine can serve as an enabler for proton‐mediated charge transport. This study demonstrates the drain‐to‐source current, PDDOS, and transmission for both structures.

Tyrosine‐rich peptide devices have been shown to function as biodegradable bimodal memory^[26]^ and humidity sensors.^[27]^ The device sensitivity and on‐off ratios can be adjusted by using different electrode materials and operational conditions. These biodegradable sensors can collect and monitor real‐time in vivo medical data. For practical applications, device structures can be scaled up to several millimeters in size since the operational mechanism relies on a peptide film rather than specific molecules. This work explores those mechanisms using models that contain five molecules due to computational limitations.

## 4 METHODS

The molecular structure and simulation environment were generated and processed using Synopsys^©^ QuantumATK^©^.[^37^, ^39^] Stick diagram figures and videos were generated by UCSF Chimera^©^.[^40^, ^41^] Density of states (DOS) calculations were based on the generalized gradient approximation (GGA) partial differential equation (PDE) built‐in exchange‐correlation algorithm with 10^−4^ tolerance. The local density approximation (LDA) basis‐set for each element was chosen to be double zeta polarized (DZP) for increased accuracy, at the expense of higher computational cost. k‐point sampling was done over a grid of 3 × 3 points in the lateral directions and 50 points along the transport direction. The interaction range was set at 20.0 Å with 1024 reciprocal points and a reciprocal energy cutoff parameter of 1250.0 Hartree. Both electrodes and central region calculators were based on a linear combination of atomic orbitals (LCAO).

## AUTHOR CONTRIBUTIONS

Dan Berco conceived the concept, developed the architectural methodologies, implemented the modeling, performed the simulations, created the visualization, and wrote the manuscript. The author would like to thank the Synopsys^©^ support team for their assistance.

## CONFLICT OF INTEREST STATEMENT

The authors declare no conflicts of interest.

## ETHICS STATEMENT

No animal or human experiments were involved in this study.

## Supporting information

Supporting Information S1

## Data Availability

The data that support the findings of this study are available on request from the corresponding author.
