# Supplementary material for: A density functional theory study of tyrosine‐proton mediated transport in Ag‐filamentary nanodevices
Source: Smart Mol. 2025 Sep 3;3(3):e70019. doi: 10.1002/smo2.70019 (PMC12483131; doi:10.1002/smo2.70019)
Supplement: Supplementary file 1 — Supporting Information S1 [file SMO2-3-e70019-s001.docx]

Supporting Information: A DFT Study of Tyrosine-Proton Mediated Transport in Ag-Filamentary Nanodevices

**Authors**

Dan Berco^1,*^

**Affiliations**

^1^ School of Electrical Engineering & Computer Science, Washington State University, 355 NE Spokane St, Pullman, WA, 99163, USA

Correspondence to:

* D.B., [danny.barkan@gmail.com](mailto:danny.barkan@gmail.com)

This work features a density functional theory study of a nanoscale neuromorphic device. The interaction between an Ag-based filamentary memristor and different proton environmental concentrations with Tyrosine molecules was studied.

# RESULTS AND DISCUSSION

**Electrostatic Difference Potential**

**Fig. S1** depicts the electrostatic drop between source and drain as a function of distance. The first building block (near the drain) is located at the zero point, while the endpoint of the fifth block (near the source) is at approximately 70 Å. **Fig. S1(a)** shows the potential distribution for the first structure, and **Fig. S1(b)** shows the second structure. The red arrows, along with numerations, indicate the effect and placement of the Tyrosine molecules and Protons. For the second structure, the hydrogen atom closer to the source is marked ‘6’. The drop is calculated for a bias potential of V_DS_ = 0 V in both cases.


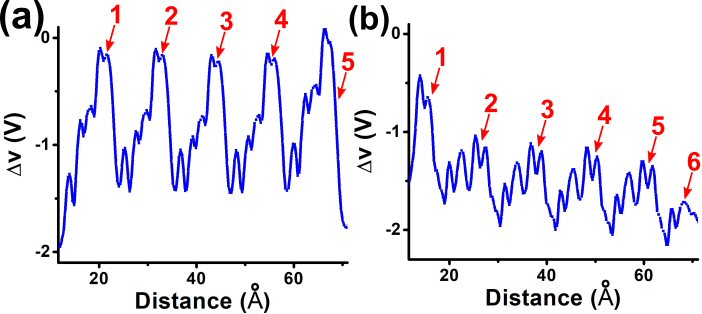


**Fig. S1.** Schematic plot of the ESD drop along the central region for both structures; the building blocks are located between 0 Å and ~70 Å in the central region; the red arrows with corresponding numbers indicate the location of individual Tyrosine-H molecules. **(a)** First structure. **(b)** Second structure.

**Transmission in Transverse K-Space**

This section details the transverse transmission components in K-space. The plots show both the total calculated density of states and a 2-D density plot of the transmission as a function of *K_A_* and *K_B_*. The drain-to-source bias *V_DS_* was modified in steps of 0.5 V, and the transmission was calculated for each step. In each case, different energy levels were selected on the density of states plot, and the corresponding components are shown beside it. **Fig. S2** to **Fig. S7** depict the transmission of the second structure for a bias of 0.0 to 2.5 V consecutively. **Fig. S8** to **Fig. S10** show the transmission of the first structure for a bias of 0.0 – 1.0 V consecutively.


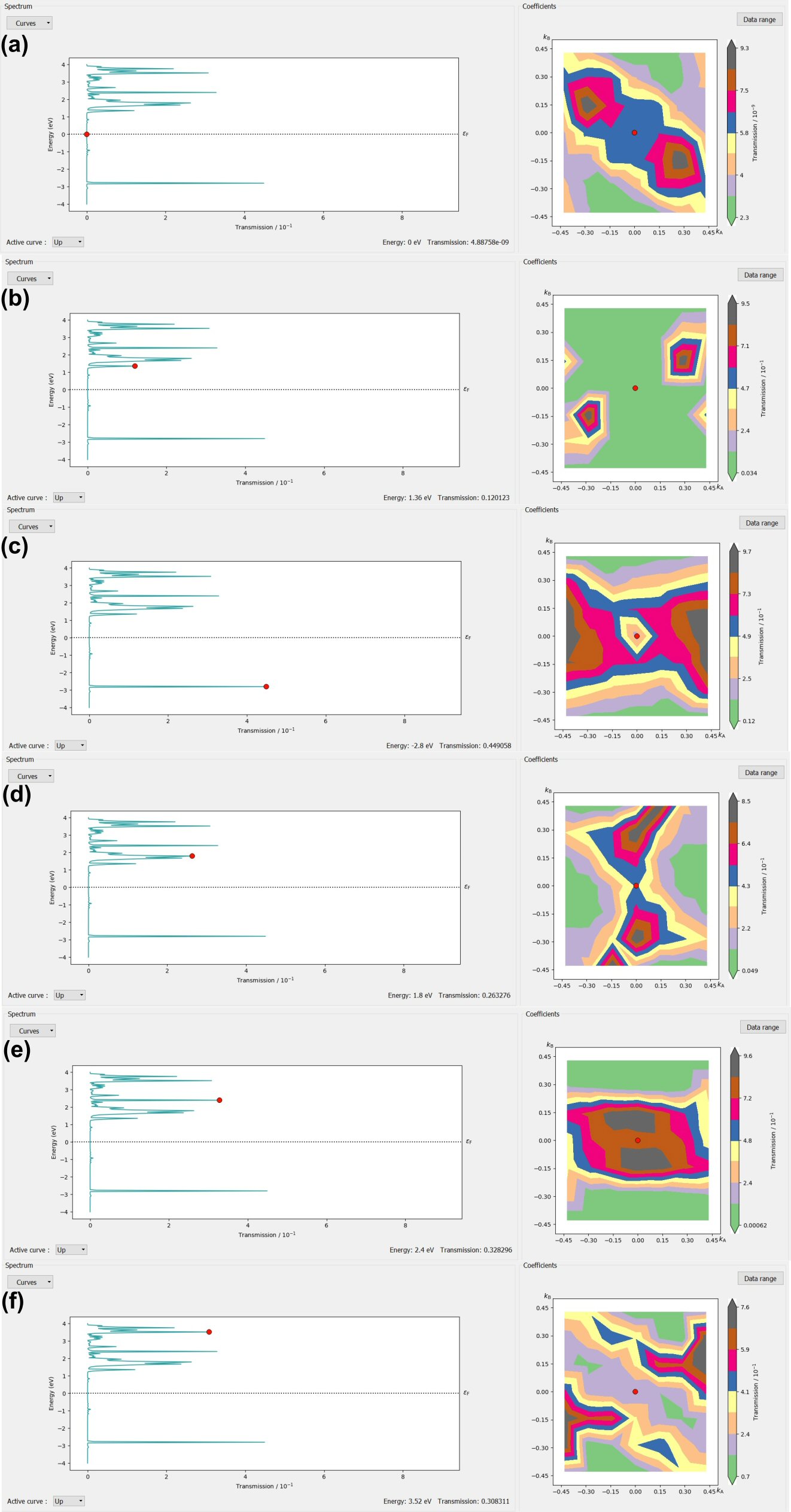


**Fig. S2.** Transmission components of the **second** structure along K_A_ and K_B_ for different energy levels and V_DS_ = 0.0 V. **(a)** E = 0 eV. **(b)** E = 1.36 eV. **(c)** E = -2.8 eV. **(d)** E = 1.8 eV. **(e)** E = 2.4 eV. **(f)** E = 3.52 eV.


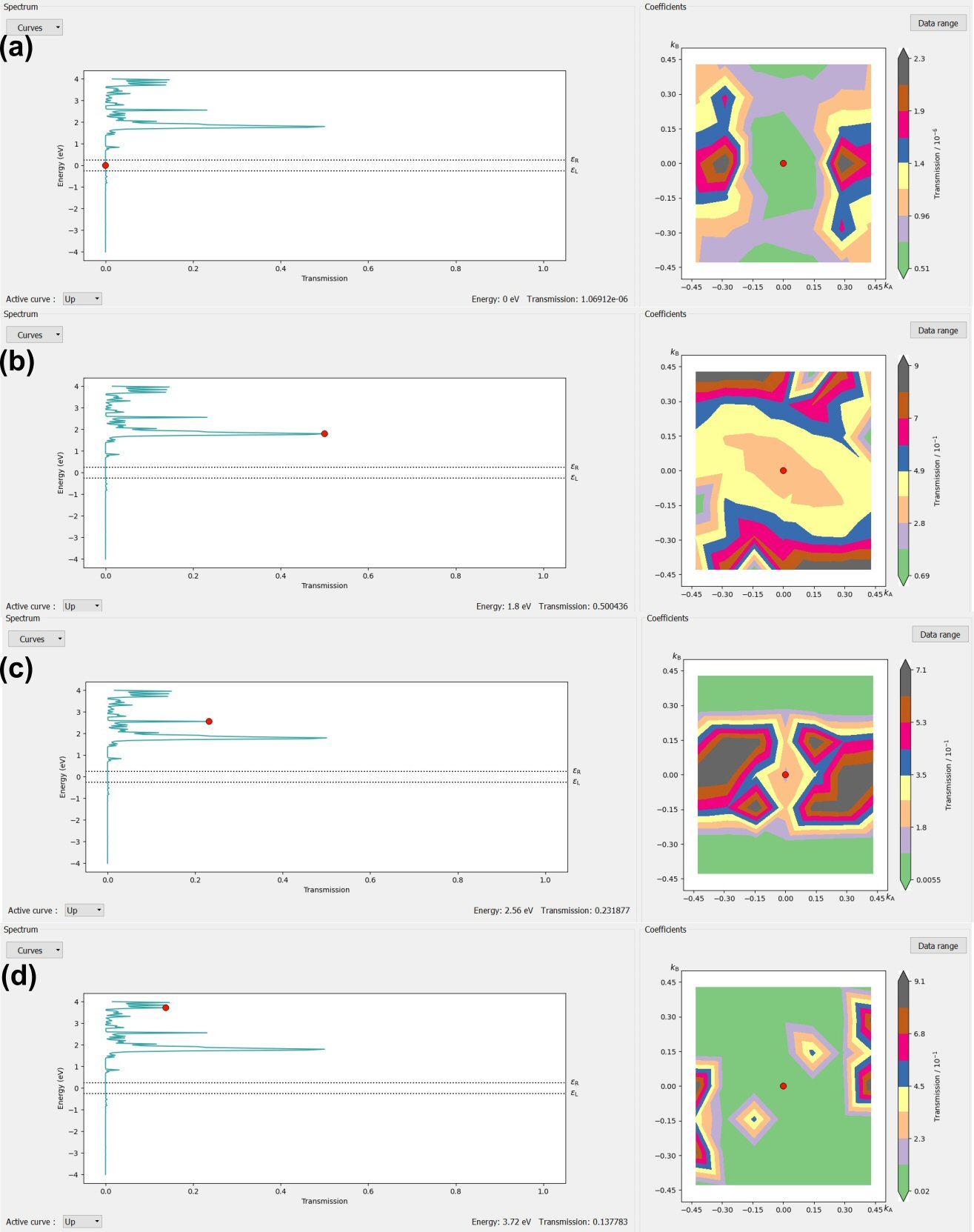


**Fig. S3.** Transmission components of the **second** structure along K_A_ and K_B_ for different energy levels and V_DS_ = 0.5 V. **(a)** E = 0 eV. **(b)** E = 1.8 eV. **(c)** E = 2.56 eV. **(d)** E = 3.72 eV.


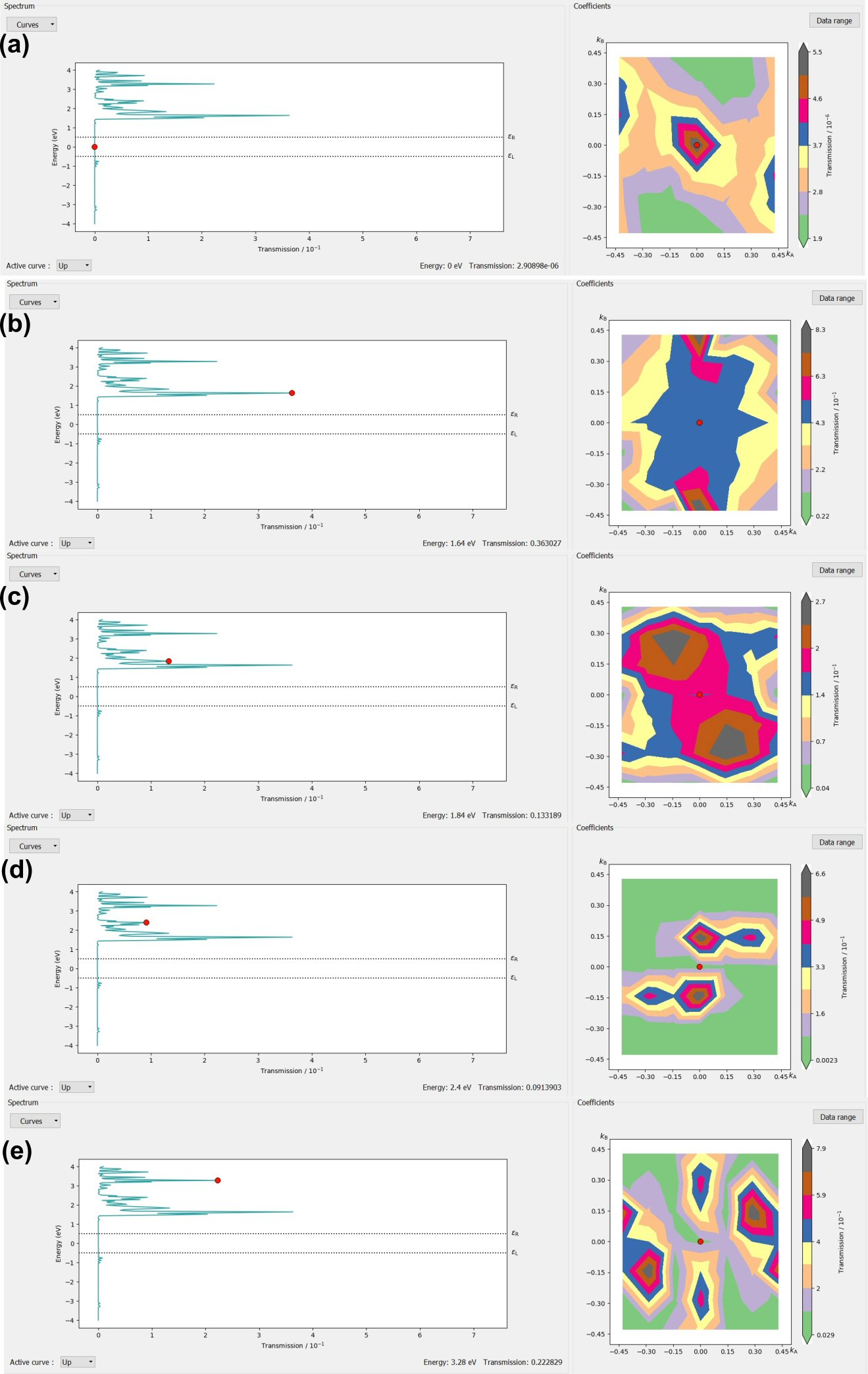


**Fig. S4.** Transmission components of the **second** structure along K_A_ and K_B_ for different energy levels and V_DS_ = 1.0 V. **(a)** E = 0 eV. **(b)** E = 1.64 eV. **(c)** E = 1.84 eV. **(d)** E = 2.4 eV. **(e)** E = 3.28 eV.


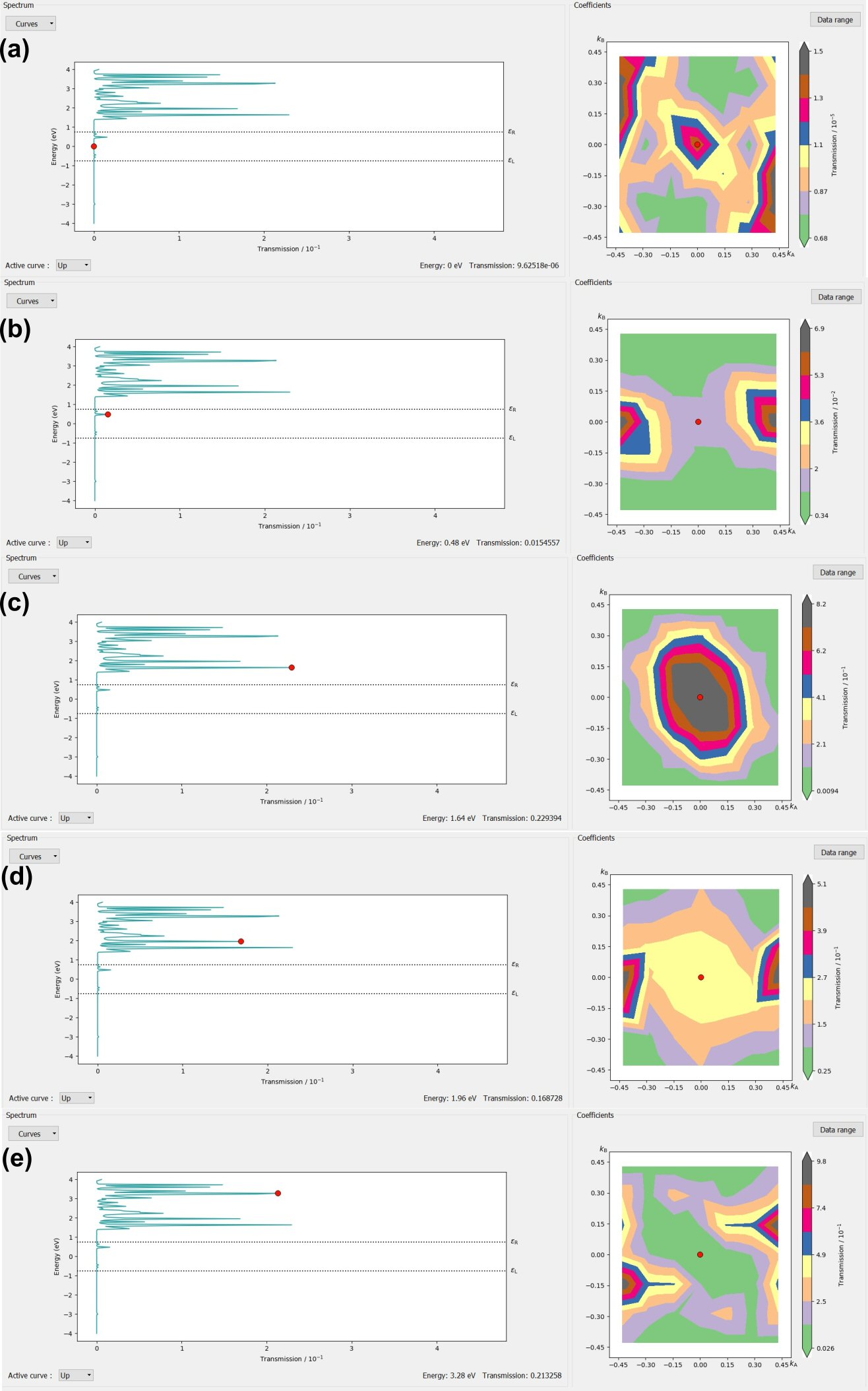


**Fig. S5**. Transmission components of the **second** structure along K_A_ and K_B_ for different energy levels and V_DS_ = 1.5 V. **(a)** E = 0 eV. **(b)** E = 0.48 eV. **(c)** E = 1.64 eV. **(d)** E = 1.96 eV. **(e)** E = 3.28 eV.

**
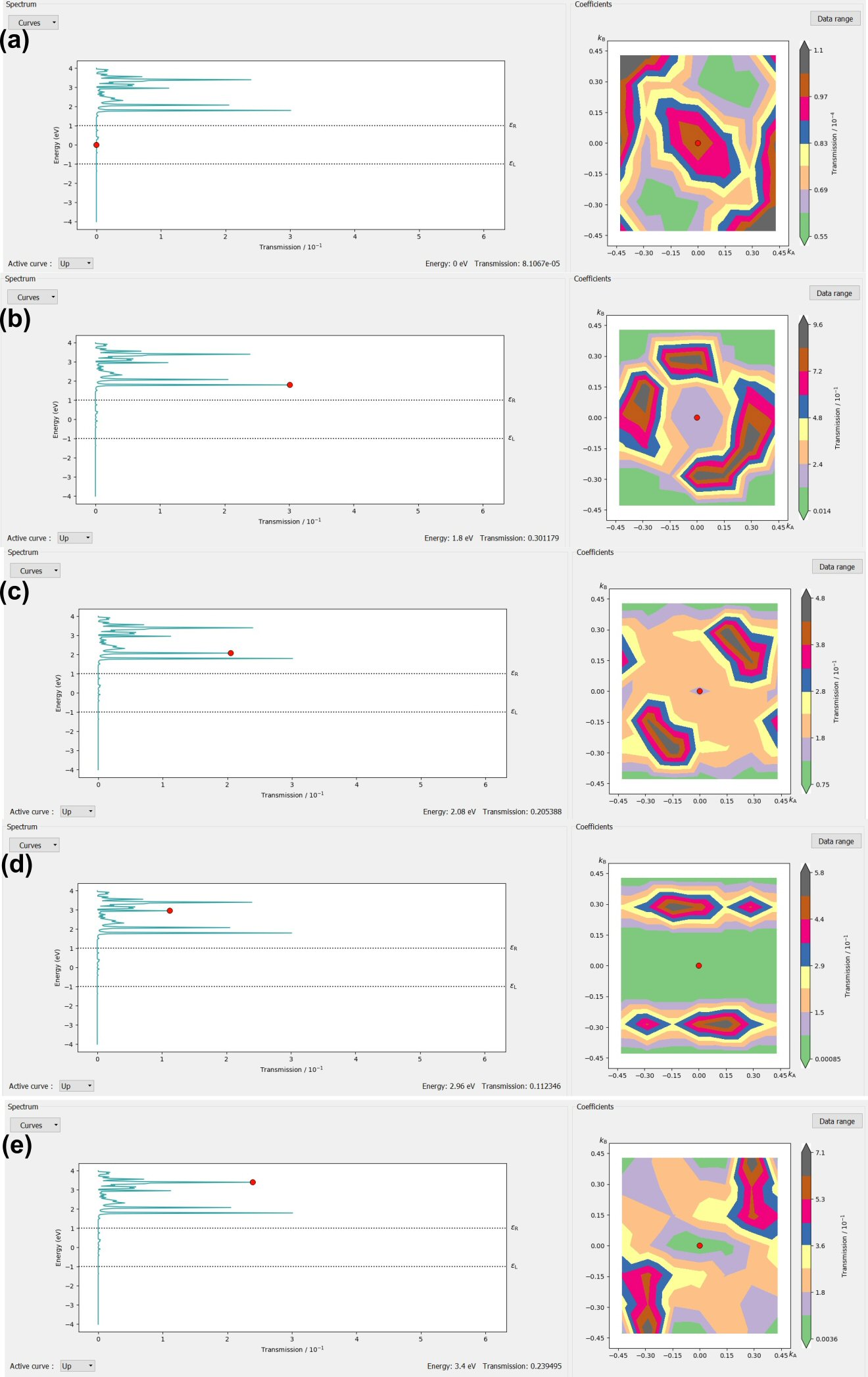
**

**Fig. S6**. Transmission components of the **second** structure along K_A_ and K_B_ for different energy levels and V_DS_ = 2.0 V. **(a)** E = 0 eV. **(b)** E = 1.8 eV. **(c)** E = 2.08 eV. **(d)** E = 2.96 eV. **(e)** E = 3.4 eV.

**
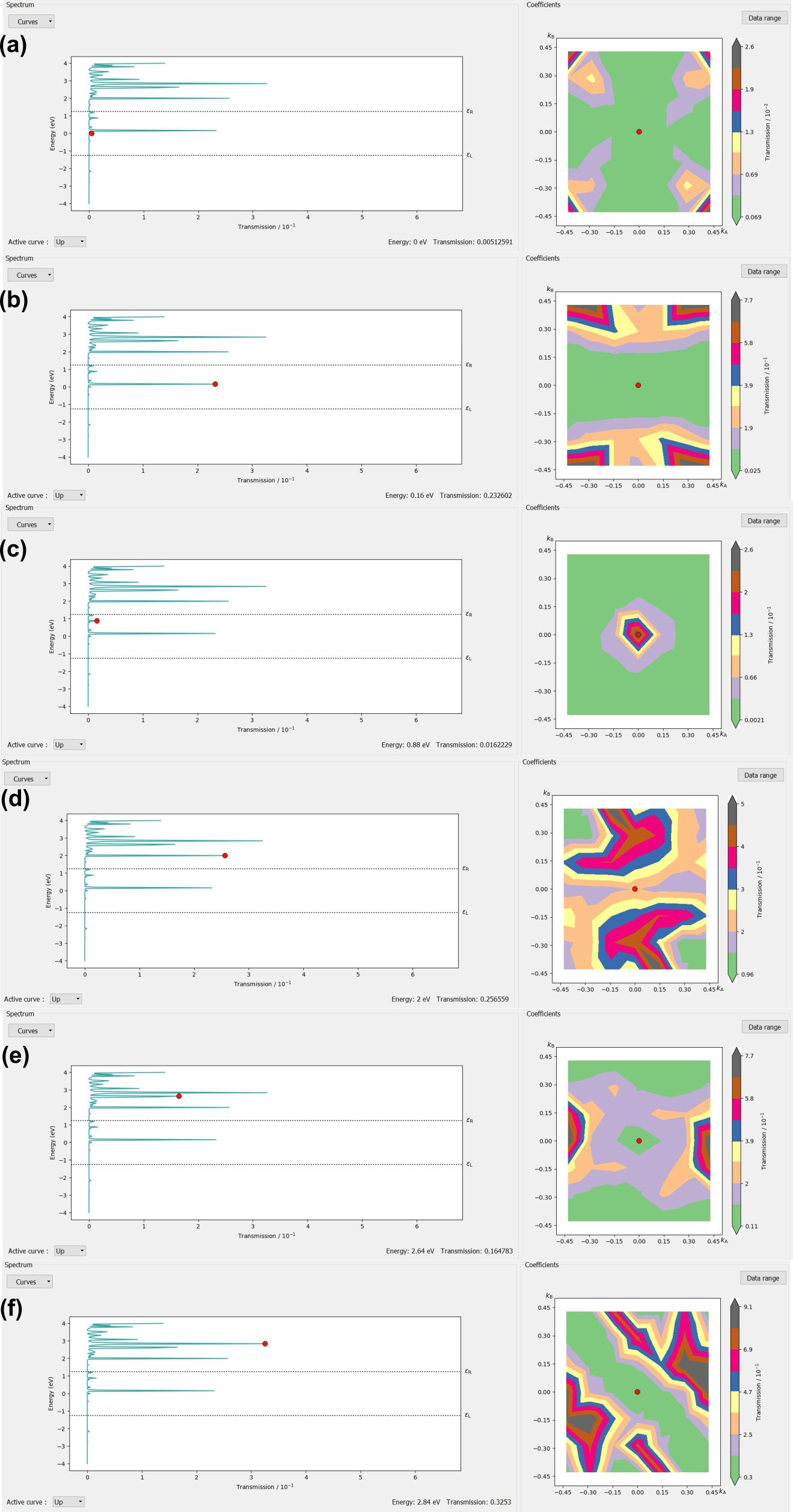
**

**Fig. S7**. Transmission components of the **second** structure along K_A_ and K_B_ for different energy levels and V_DS_ = 2.5 V. **(a)** E = 0 eV. **(b)** E = 0.36 eV. **(c)** E = 0.88 eV. **(d)** E = 2.0 eV. **(e)** E = 2.64 eV. **(f)** E = 2.84 eV.

**
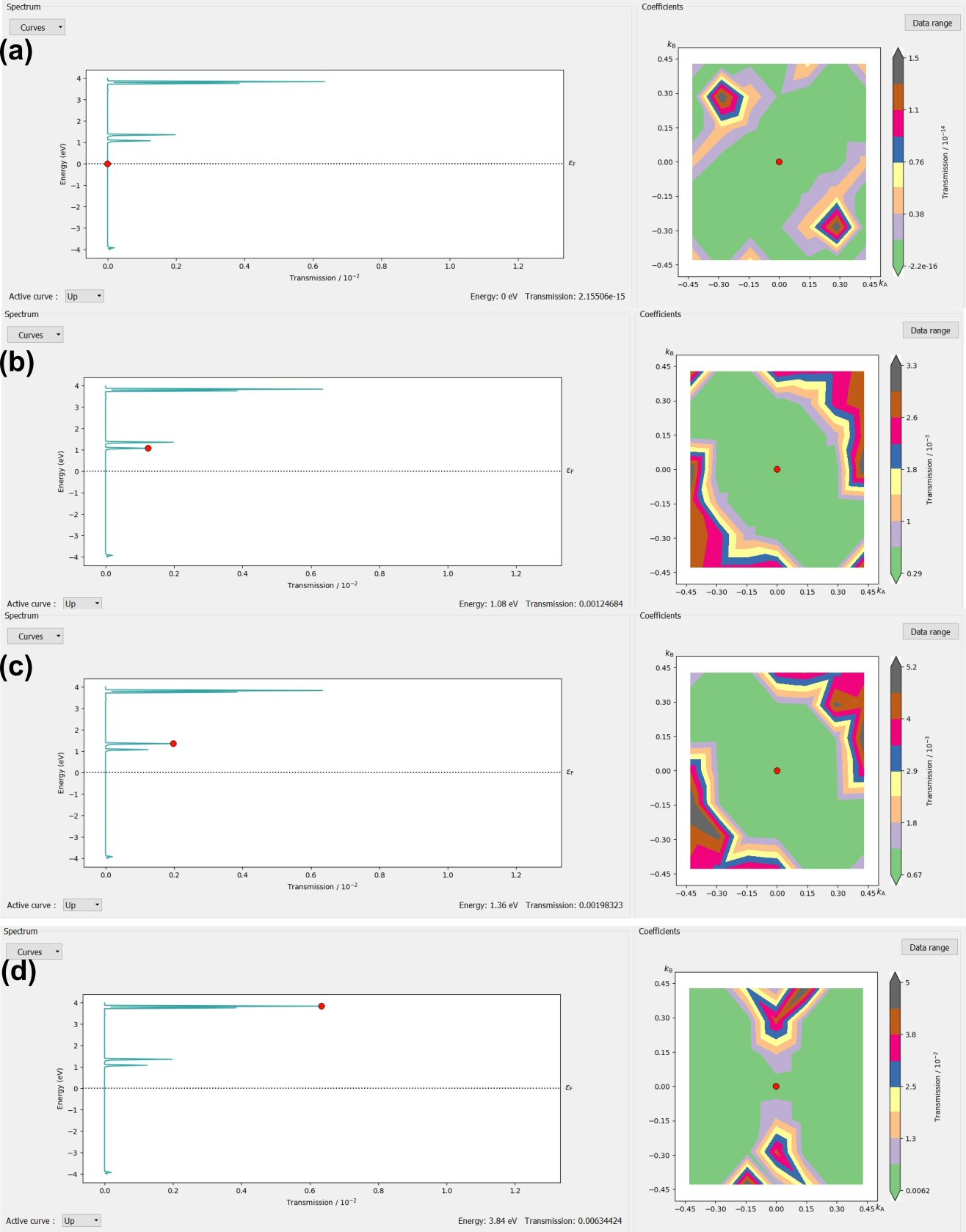
**

**Fig. S8**. Transmission components of the **first** structure along K_A_ and K_B_ for different energy levels and V_DS_ = 0.0 V. **(a)** E = 0 eV. **(b)** E = 1.08 eV. **(c)** E = 1.36 eV. **(d)** E = 3.84 eV.

**
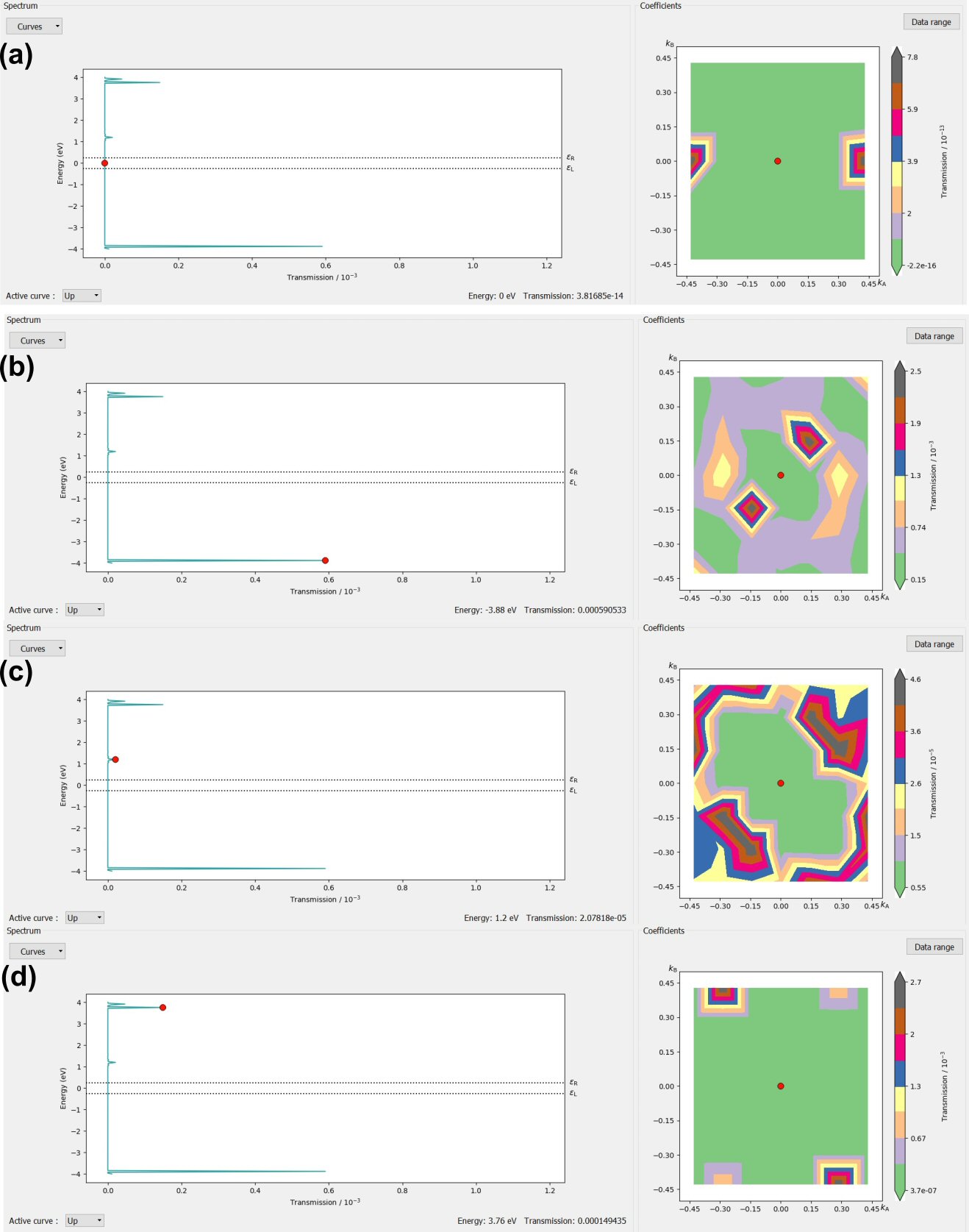
**

**Fig. S9**. Transmission components of the **first** structure along K_A_ and K_B_ for different energy levels and V_DS_ = 0.5 V. **(a)** E = 0 eV. **(b)** E = -3.88 eV. **(c)** E = 1.2 eV. **(d)** E = 3.76 eV.

**
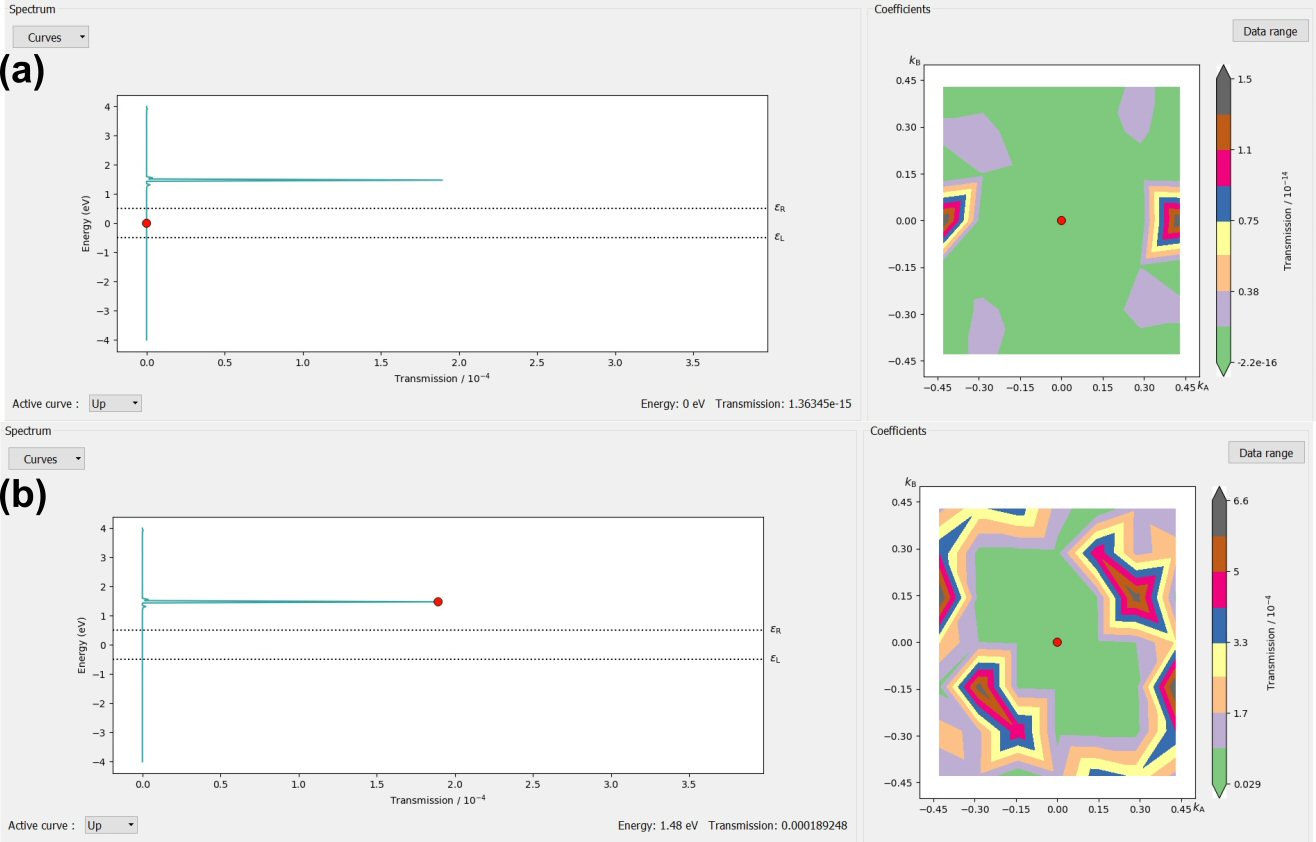
**

**Fig. S10**. Transmission components of the **first** structure along K_A_ and K_B_ for different energy levels and V_DS_ = 1.0 V. **(a)** E = 0 eV. **(b)** E = 1.48 eV.

# METHODS

The molecular structure and simulation environment were generated and processed using Synopsys© QuantumATK^© 1, 2^. Stick diagram figures and videos were generated with UCSF Chimera^© 3, 4^.

# AUTHOR INFORMATION

**Corresponding Authors:**

E-mail (D. Berco): [danny.barkan@gmail.com](mailto:danny.barkan@gmail.com)

**ORCID**

Dan Berco: 0000-0003-2902-1422

# ACKNOWLEDGMENTS

**Author contributions:** D.B conceived the concept, developed the architectural methodologies, implemented the modeling, performed the simulations, created the visualization, and wrote the manuscript.

**Competing interests:** The authors declare no conflict of interest.

# REFERENCES

1. Synopsys QuantumATK ver. W-2023.09 <https://www.synopsys.com/quantumatk>
2. S. Smidstrup, T. Markussen, P. Vancraeyveld, J. Wellendorff, J. Schneider, T. Gunst, B. Verstichel, D. Stradi, P. A. Khomyakov, U. G. Vej-Hansen, and M. E. Lee, QuantumATK: an integrated platform of electronic and atomic-scale modelling tools. Journal of Physics: Condensed Matter, 32(1), p. 015901 (2019).
3. E. F. Pettersen, T. D. Goddard, C. C. Huang, G. S. Couch, D. M. Greenblatt, E. C. Meng, and T. E. Ferrin, UCSF Chimera-a visualization system for exploratory research and analysis. J Comp. Chem, 25(13), p. 1605-12 (2004).
4. UCSF Chimera version 1.16 <https://www.cgl.ucsf.edu/chimera/>
